# A review of glioblastoma tumors with primitive neuronal component and a case report of a patient with this uncommon tumor at a rare location

**DOI:** 10.1002/ccr3.3228

**Published:** 2020-08-12

**Authors:** Azin Shayganfar, Shadi Ebrahimian, Parvin Mahzouni, Fattane Shirani, Marzieh Aalinezhad

**Affiliations:** ^1^ Department of Radiology Isfahan University of Medical Sciences Isfahan Iran; ^2^ Department of Pathology Isfahan University of Medical Sciences Isfahan Iran

**Keywords:** glioblastoma, infratentorial, primitive neuronal component, tumor

## Abstract

Glioblastoma with primitive neuronal component should be considered as a differential diagnosis of infratentorial tumors.

## Graphical abstract

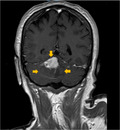

## 1 INTRODUCTION

Glioblastoma with primitive neuronal component (GBM‐PNC) is a new pattern of glioblastoma introduced in 2016. The most common location of this tumor is the temporal lobe. There are few cases of infratentorial GBM‐PNC reported. We reported a patient with cerebellar GBM‐PNC, a very rare location of this tumor.

Glioblastoma multiform (GBM) is the most frequent malignant astrocytoma presented mostly among adults. Although radiation therapy and chemotherapy are used as standard treatments, most of the GBMs are resistant to treatment and have a poor prognosis.^1^ In the world health organization classification of central nervous system (2016), glioblastomas are divided into IDH‐wild type, IDH‐mutant, NOS glioblastoma, and GBM‐PNC. GBM‐PNC is a newly introduced pattern of glioblastoma that often arises from a preexisting high‐grade glioma. These tumors have features of both malignant glioma and primitive neuroectodermal tumor (PNET).^2^

Glioblastoma with primitive neuronal component is an aggressive tumor with a short survival rate because of the invasion and metastases to the surrounding tissues and entire neuroaxis.^3^, ^4^ Radiotherapy and chemotherapy after total resection of the tumor are the therapies. The prognosis of the disease is related to the craniospinal metastasis or recurrence of the tumor.^5^ Early detection of the tumor can improve the prognosis of the disease. The physician's awareness of this tumor can lead to the early diagnosis, management, and decreasing the morbidity and mortality rate. In this study, therefore, a case of this rare tumor is reported at a rare location, and a literature review is presented on the epidemiology, pathologic and radiologic features, treatment, and prognosis of this rare tumor.

## 2 CASE HISTORY

**FIGURE 1:**
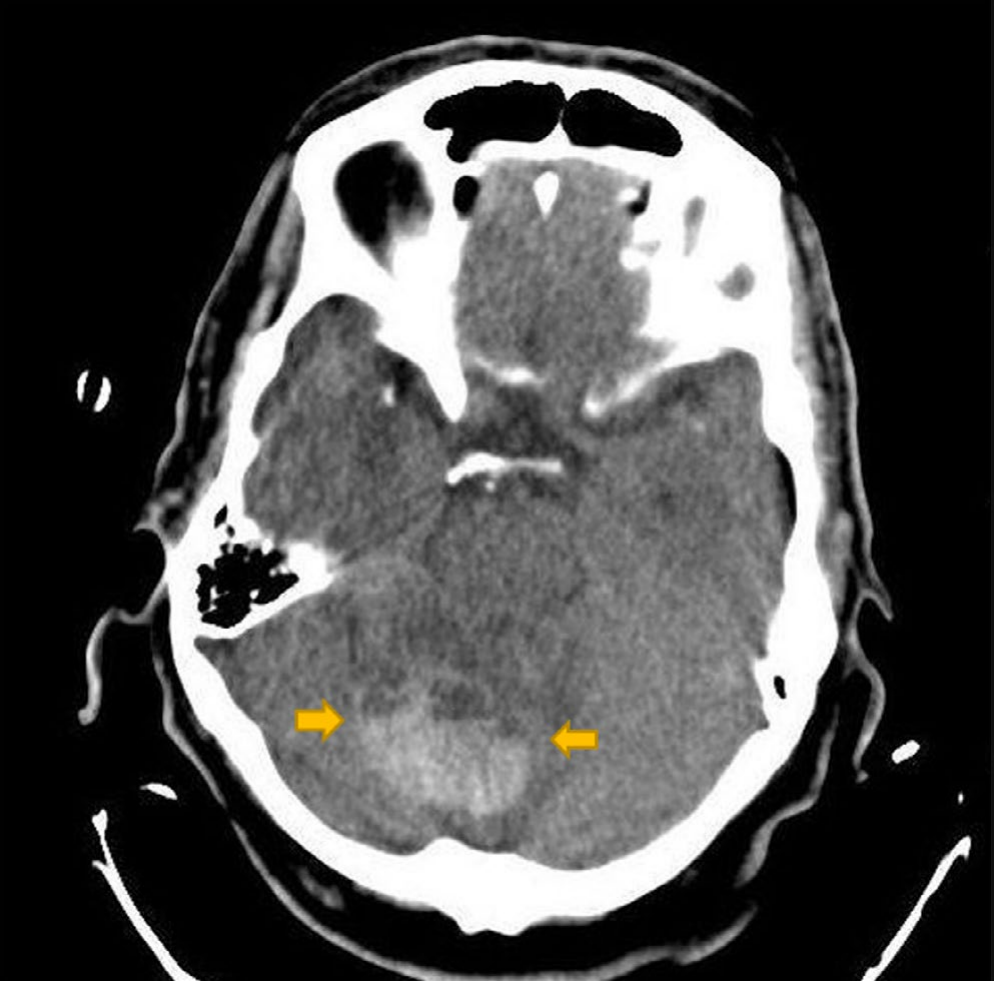
Right cerebellar hemorrhagic mass with peripheral edema in CT scan without contrast

**FIGURE 2:**
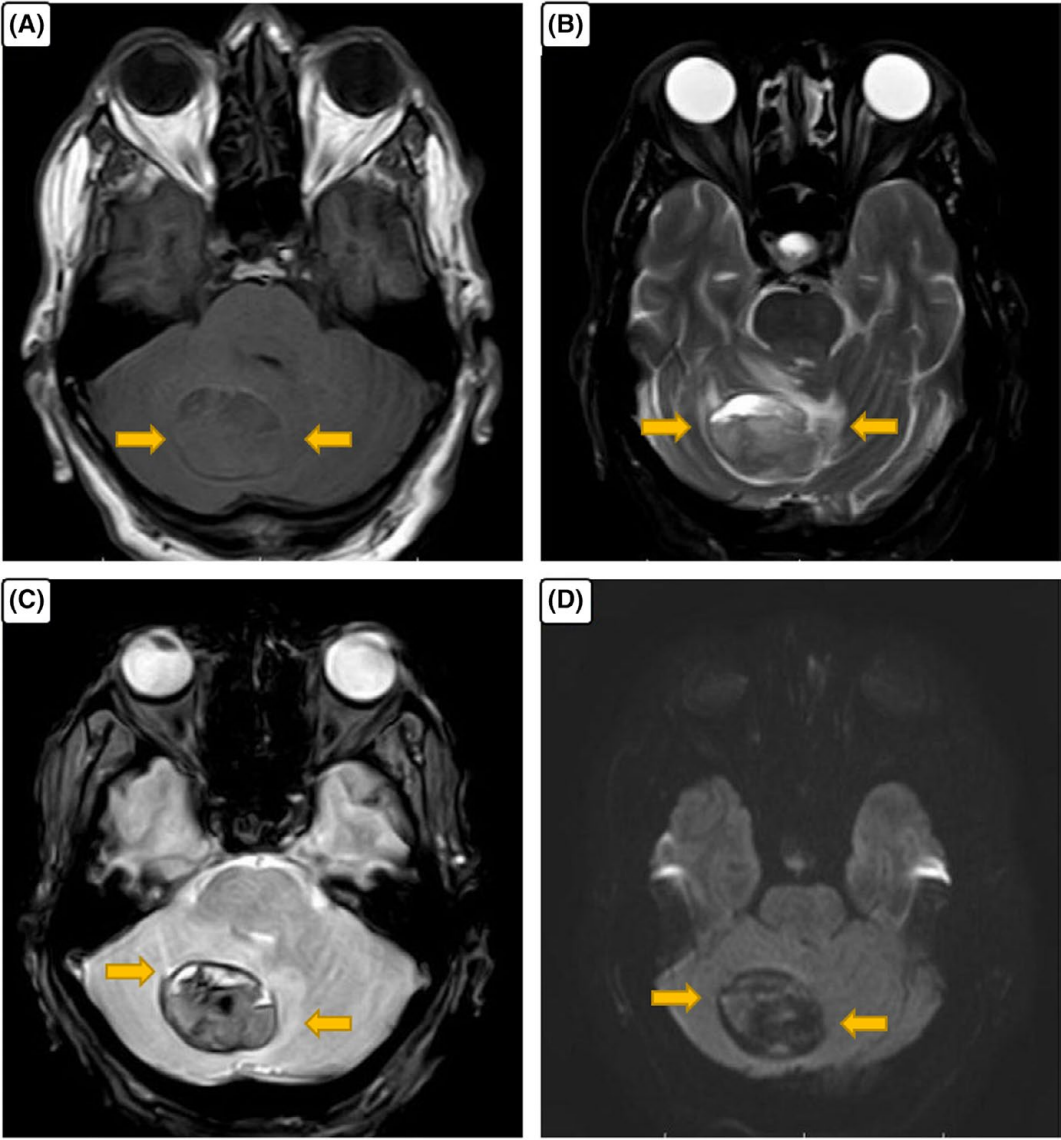
A, B, A right cerebellar mass contain fluid‐fluid level with heterogeneous low to intermediate signal intensity in T1W and intermediate to high signal intensity in T2W; C, Dark signal intensity in T2*sequence with blooming effect representing acute hemorrhage; D, No diffusion restriction is present in DWI sequence

**FIGURE 3:**
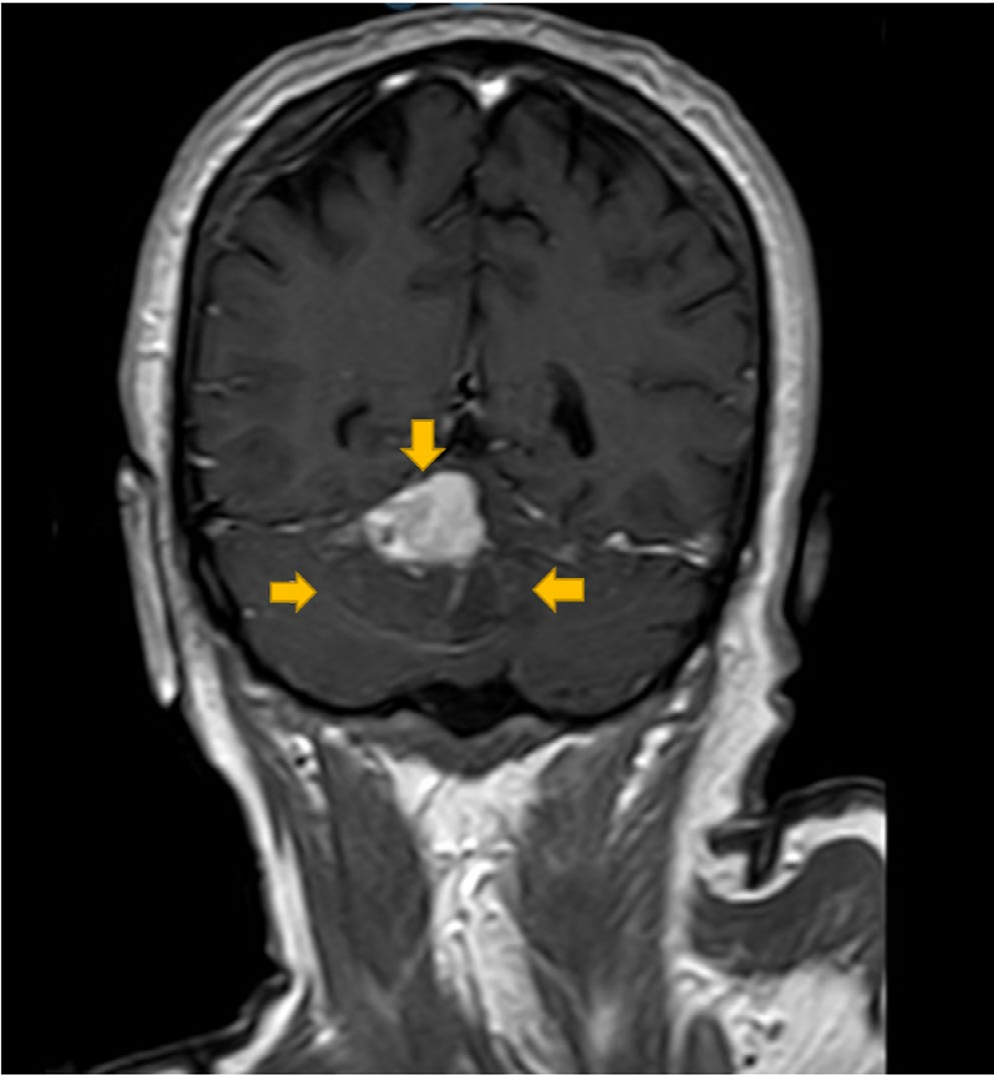
Intense enhancement of solid part in the most upper component in postcontrast T1W

A 71‐year‐old man presented with progressive true vertigo, nausea, and vomiting. Brain computed tomography revealed a circumscribed intraaxial hemorrhagic mass in the center of the right cerebellar hemisphere (Figure 1). The patient underwent a brain MRI with and without contrast. MR images were obtained using a 1.5 Tesla MR scanner (Ingenia, Philips). Brain MRI with multiplane images in different pulse sequences revealed a mass placed in the center of the right cerebellar hemisphere and superior vermis with a fluid‐fluid level and a signal compatible with acute hemorrhage. In T1‐weighted images, intense homogenous enhancement of solid part located in the upper section was revealed after contrast administration. The findings were compatible with acute hemorrhagic cystic cerebellar mass (Figures 2 and 3). A mild noncommunicating ventriculomegaly was developed secondary to the mass effect. Major intracranial vessels, basal ganglia, brain stem, optic nerve, facial nerve, and vestibule‐cochlear nerve complex, pituitary gland, and orbits were all normal. The patient underwent posterior fossa craniotomy, and the excised 5.5 × 4 × 0.5 cm soft tumor consisted of multiple soft gray tissues. Microscopically, the cerebellum was infiltrated by a tumor consisting of two parts. Pathology revealed a neoplastic proliferation of small undifferentiated cells with oval hyperchromic nuclei and scant infiltrated cytoplasm, along with Oligodendroglial cells with round nuclei, clear halo, and glomeruloid formation.

**FIGURE 4:**
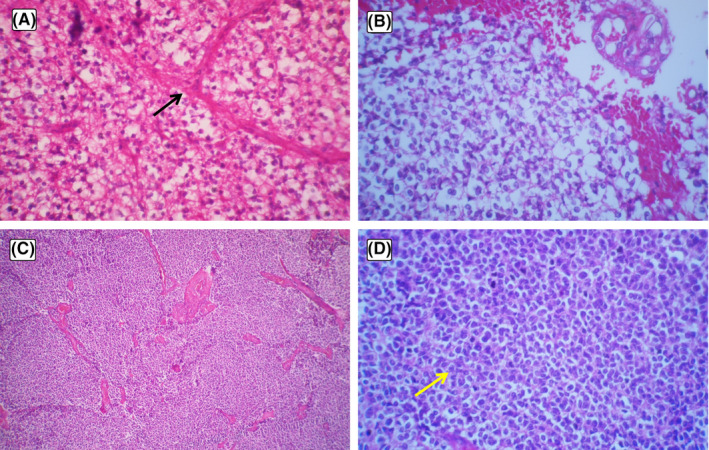
Low power: The oligodendroglial component shows cells with round nuclei, clear halo, and branched capillary vessel (black arrow) (A); High power: The oligodendroglial component with glomeruloid formation (B); Low power: The primitive neuroectodermal component shows highly cellular tumor with small undifferentiated cells (C); High power: The primitive neuroectodermal component demonstrates small undifferentiated cells with scant cytoplasm and hyperchromatic oval nuclei (yellow arrow) (D)

Vascular glomeruloid formation and perivascular pseudorosettes were also detected in PNET area. Immunostains showed that the tumor was immunopositive for Synaptophysin in PNET components and GFAP positive in glioblastoma areas. CD20, LCA, CK, and TTF1 were negative. The tumor was diagnosed as GBM‐PNC (Figure 4).

## 3 DISCUSSION

Glioblastoma with primitive neuronal component is a very rare tumor accounting for 0.5% of all GBMs, being more common among adults. However, some cases have been reported in children aged 12 and 17 years. Moreover, Perry et al^3^ reported a median age of 54 years, ranging from 21 to 80 years.

Although the prognosis of GBM‐PNC is poor, long‐term survival is variable. In the study on 53 cases of GBM‐PNC, the median survival rate was 9.1 months from the first diagnosis and ranged from less than a month to 33 years.^3^ However, another study on 40 cases revealed a mean survival of 44 months.^6^ The overall survival in four patients with GBM‐PNC diagnosis was reported from 12 to 24 months from the initial diagnosis. Longer survival rate was seen in patients receiving more aggressive treatments.^7^ A longer survival rate was reported by Chen in a 23‐year‐old female who survived for 9 years after the tumor diagnosis.^8^

Histologically, The GBM‐PNC consists of two components. The glial component consists of astrocytes with abundant eosinophilic cytoplasm and large vesicular nuclei, mitosis, necrosis, and microvascular proliferation on the fibrillary background. The hypercellular area of small cells with a narrow cytoplasm, hyperchromic nuclei, pseudorosettes forming, and a multifocal distribution is the histologic features of the PNC.^9^

Glioblastoma with oligodendroglial differentiation (4%‐20% of GBMs) composed of foci with clear cytoplasm, round nuclei, branched capillary vessels, and calcification. The presence of oligodendroglial component associated with co‐deletion of chromosome 1p and 19q appears to be an important prognostic factor.^2^

The diagnosis of GBM‐PNC is confirmed using immunohistochemistry. The glial component of the tumor is usually positive for GFAP, S100, OLIG2, and vimentin, while the PNC always expressed the synaptophysin and is negative for glial markers. CD56 is reported as a positive marker in both components.^3^, ^10^, ^11^ The tumor may also manifest IDH1 mutation. Patients with an IDH1 mutation are more likely to have secondary GBM and a better prognosis.^8^, ^11^

The metastasis is not common among patients with GBM tumor, and it accounts for <2% of the GBMs. The spinal metastasis is very rare in GBMs. However, GBM‐PNC has a high tendency to spinal metastasis and cerebrospinal fluid dissemination.^12^, ^13^ Although spinal metastasis is common in patients with GBM‐PNC, some other extracranial metastases to lung and bone have been reported.^13^, ^14^

Temozolomide is the treatment of GBM, while platinum‐based chemotherapy is effective on PNETs. However, the treatment of GBM‐PNC is still a challenge due to few available data on therapeutic approaches. However, in a review of the literature on GBM‐PNC treatment, Prelaj et al^15^ suggested an aggressive treatment using a multimodal approach including surgery, chemoradiotherapy, and platinum‐based chemotherapy as an efficient therapeutic approach. Also, a mutation in PTEN‐PI3K pathway was seen in a recent study by Xu et al^16^ on whole‐exome sequencing of GBM‐PNC, suggesting the use of targeted therapeutic agents including PI3K inhibitors, mTORC1 inhibitors, or dual PI3K/mTOR inhibitors as a future treatment of GBM‐PNC.

The tumor is most likely diagnosed using imaging in a patient with symptoms of CNS involvement. Reports on radiologic features of GBM‐PNC are very rare. Ali et al studied on radiologic features of GBM‐PNC tumors in nine patients and compared them with conventional GBM. They found a reduced Apparent Diffusion Coefficient (ADC) in GBM‐PNC tumors compared to conventional GBM, suggesting that significantly reduced diffusion of a tumor on MRI should be considered as a diagnostic radiologic feature of GBM‐PNC.^17^ Their results were similar to another study on 53 patients with GBM‐PNC lesions, reporting reduced diffusion in these tumors. The observed restricted diffusion is because of the increased cellularity in the PNET component, which leads to a decrease in water motion and diffusion.^11^ In 53 patients with GBM‐PNC, imaging features revealed cerebral masses with heterogeneous or ring enhancement which were not different from other GBMs or CNS PNETS.^3^ Other important radiologic features of GBM‐PNC are a well‐circumscribed tumor with significant mass effect and peripheral edema. Intralesional hemorrhage, necrosis, and cystic areas were also reported elsewhere.^9^ In our case, a cystic hemorrhagic mass was detected in MRI sequences, which was compatible with the radiologic features reported in the literature.

The most frequent location of GBM‐PNC is the temporal lobe occurring in 52% of cases.^18^ Of 10 cases of GBM with PNET components reported by Song et al, all tumors were located supratentorial, mostly in the temporal and frontal lobes. The MRI findings in these cases were similar to conventional GBMs, with DWI showing focal or extensive and obvious restricted diffusion.^11^ However, out of the nine patients reported by Ali et al,^17^ the frontal lobe was the most frequently involved one followed by temporal, parietal, and occipital lobes.

There are multiple cases of GBM‐PNC reported in the literature.^19^, ^20^, ^21^ However, GBM‐PNCs with infratentorial location were reported in a few cases of GBM‐PNC.^7^, ^13^ Infratentorial tumors are more common among children and account for 48% of primary intracranial tumors. In adults, about 15%‐20% of brain tumors are in posterior fossa.^22^ The most common type of posterior fossa neoplasms in adults are metastases accounting for 20% of brain metastasis.^23^, ^24^ Glioblastoma is more likely to develop in supratentorial region and cerebral hemispheres. Cerebellum is an uncommon location for GBM and GBM subtypes. Although the cerebellum is 10% of total brain weight, only 1% of GBMs arise from there.^25^, ^26^ In a study that reported five cases of GBM‐PNC lesions, a cerebellar lesion was detected only in a 71‐year‐old man, looked multiloculated with cystic, and hemorrhagic necrosis and irregular rim enhancement.^7^ In our case, the reported 71‐year‐old man presented with vertigo and an infratentorial cerebellar GBM‐PNC. This case is the second cerebellar GBM‐PNC reported in the literature.

In our case, a GBM‐PNC was observed with MRI features similar to previously reported tumors, but at a rare location. This report suggests that although infratentorial GBM‐PNC tumors are rare, they should be considered as a differential diagnosis in any infratentorial tumor.

## CONFLICT OF INTEREST

None declared.

## AUTHOR CONTRIBUTION

AS, SE, PM, FS, MA: contributed in data gathering, writing the manuscript, and all read the final version of the manuscript and approved that.
